# Heterogeneous expression of the collagen receptor DDR1 in chronic lymphocytic leukaemia and correlation with progression

**DOI:** 10.1038/bcj.2016.121

**Published:** 2017-01-06

**Authors:** G Barisione, M Fabbi, G Cutrona, L De Cecco, S Zupo, B Leitinger, M Gentile, M Manzoni, A Neri, F Morabito, M Ferrarini, S Ferrini

**Affiliations:** 1 IRCCS AOU San Martino-IST Istituto Nazionale per la Ricerca sul Cancro, Genoa, Italy; 2 Fondazione IRCCS Istituto Nazionale Tumori, Milan, Italy; 3 Section of Molecular Medicine, National Heart and Lung Institute, Imperial College London, UK; 4 Hematology Unit Azienda Ospedaliera of Cosenza, Cosenza, Italy; 5 Department of Oncology and Hemato-oncology, University of Milan, Milan, Italy; 6 Hematology Unit, Fondazione Cà Granda IRCCS Ospedale Maggiore Policlinico, Milan, Italy; 7 Biotechnology Research Unit, Aprigliano, ASP of Cosenza, Cosenza, Italy

Discoidin domain receptor (DDR)1 is a tyrosine kinase-receptor, which is activated by various types of collagens.^1^ Several studies showed DDR1 expression in human cancers, and reported its role in cell proliferation, epithelial to mesenchymal transition, migration and invasiveness.^2^ In addition, high DDR1 expression has been related to adverse prognosis in some human solid tumours. As far as haematological malignancies are concerned, *DDR1* gene is highly expressed in adult B-ALL cases without ALL1/AF4 and E2A/PBX1 molecular rearrangements, as well as in B-cell receptor/ABL-positive cases.^3^ Also, acute myeloid leukaemia (AML) blasts express DDR1, which provides a supportive stimulus in the bone marrow microenvironment.^4^ Moreover, activation of DDR1 protects Hodgkin lymphoma cells from apoptosis. Although Hodgkin and Reed–Sternberg cells express DDR1, it is not detected in their normal counterpart, the germinal centre B cells of reactive tonsils.^5^ The microenvironment of the bone marrow and secondary lymphoid organs has an essential role in chronic lymphocytic leukemia (CLL) pathogenesis and resistance to treatment.^6^ Indeed, stimuli deriving from the B-cell receptor, cell-to-cell contacts with nurse-like cells or activated T cells, and several chemokines and cytokines promote CLL proliferation and survival.^6, 7^ In a previous study, we reported that IL-21, which regulates CLL B-cell survival in a context-dependent fashion, modulates the expression of several mRNAs, among which *DDR1* was one of the most downregulated genes.^8^ These data suggested that DDR1 may be expressed in CLL cells but, to the best of our knowledge, no other studies have specifically addressed this issue. Since CLL is a clinically heterogeneous disease, here we studied the expression of *DDR1* gene in independent retrospective series of CLL in relationship to time to first treatment (TTFT), known prognostic markers, and miRNA expression.

To analyse *DDR1* mRNA expression we used three public data sets of CLL gene expression.^9, 10, 11^ The raw gene and miRNA expression data were retrieved from the NCBI Gene Expression Omnibus repository (http://www.ncbi.nlm.nih.gov/geo/) through GEO Series accession numbers GSE22762 (GPL570), GSE39671 and GSE40571. The raw intensity expression values were processed by Robust Multi-array Average procedure with the re-annotated Chip Definition Files from BrainArray libraries version 18.0.0 available at http://brainarray.mbni.med.umich.edu. The statistical procedures were standard functions in base R package (Pearson's product–moment correlations and Wilcoxon rank-sum tests). TTFT analysis was performed using the Kaplan–Meier method. A value of *P*<0.05 was considered significant. To test DDR1 protein expression, blood samples were obtained with approval of the Institutional Review Board and informed written consent of the patients, in accordance with the declaration of Helsinki. Indirect immunofluorescence with anti-DDR1 murine IgG1 mAb (clone 7A9)^12^ and Western blot analysis of DDR1 expression were performed as detailed in the Supplementary information.

Since DDR1 expression correlates with disease progression in different types of cancers, we first analysed *DDR1* mRNA in three public data sets of CLL gene expression. The analysis of these CLL cohorts, stratified according to *DDR1* cut-off levels, showed that high *DDR1* gene expression correlates with a shorter TTFT (Figures 1a–c). Next, we analysed whether *DDR1* expression had any relationship with known prognostic markers of CLL, such as ZAP70, CD38 and *IGVH* mutational status.^13^ A significant, albeit borderline, correlation between *DDR1* and *ZAP70* mRNA levels was found in the GSE22762-GPL570 (*r*=0.24, *P*=0.0064), GSE39671 (*r*=0.31, *P*=1.6e-04) and GSE40571 (*r*=0.29, *P*=4.674e-06) data sets. Concerning CD38 expression, a weak correlation was found in the GSE39671 (*r*=0.19, *P*=0.015) and in the GSE40571 (*r*=0.17, *P*=0.0098) data sets. Furthermore, cases with high ZAP70 protein expression or with unmutated *IGVH* genes showed higher *DDR1* gene expression, in the GSE40571 data set (Figures 1d and e). To further investigate the DDR1 surface expression and its molecular form we performed immunofluorescence and western blot analysis on CLL cells from a small cohort (Supplementary Table S1). Peripheral blood leukaemia cells express surface DDR1 at variable levels, in different CLL cases, as detected by immunofluorescence (representative cases are shown in Figure 2a and Supplementary Figure S1A). Out of 34 CLL tested (Supplementary Table S2), 11 showed a DDR1 low/negative phenotype (<30% of DDR1^+^ cells), 13 were DDR1 bright (>65%) and 10 showed intermediate levels, indicating heterogeneity. Of note, DDR1 is virtually absent in circulating mature B cells (CD20^+^) from healthy donors, whereas DDR1 and CD20 double staining confirmed expression on leukaemia B cells (Supplementary Figure S1B). Western blot analysis with an anti-DDR1 antibody showed a predominant band of ~130 kDa, under reducing conditions, which displayed variable intensity in the different CLL samples (figure 2B and Supplementary Figure S2). As control, the K562 erythroleukaemia cell line, known to express full length DDR1,^4^ showed a band of the same size. On the basis of the correlation of ZAP70 and DDR1 gene expression, we verified whether such relationship also exists at the protein level. To this end, we re-probed the same blots with an anti-ZAP70 antibody. Densitometry analyses, normalised to the β-actin content, confirmed the correlation of ZAP70 and DDR1 at the protein level, in a small independent cohort (*n*=12, *r*=0.7413, CI: 0.2733–0.9255, *P*=0.0078; Figure 2c). Since DDR1 expression in activated T cells has been related to ERK1/2 phosphorylation,^14^ we also analysed phosphorylated ERK1/2, but no correlation with DDR1 was found (Figure 2c and Supplementary Figure S2). Finally, our previous data indicated that miR663b may downregulate DDR1 expression in response to IL-21 stimulation of CLL cells, *in vitro*.^8^ However, we could not find a significant anti-correlation between miR663b and *DDR1* (data not shown) by an integrated analysis of gene expression profiling and miRNAome in the proprietary database GSE40571.^11^ Also, miR199b-5p, which was reported to downregulate DDR1 expression in AML,^4^ showed a good, albeit non-significant, reverse correlation with *DDR1* mRNA levels (data not shown).

In this study, we show for the first time, that the tyrosine kinase DDR1 is expressed at the cell surface of circulating B-CLL cells, although with a remarkable heterogeneity among individual cases. In addition, *DDR1* gene expression correlates with that of the ZAP70 tyrosine kinase and with the *IGVH* mutational status, which are regarded as prognostic markers in CLL.^13^ Accordingly, high *DDR1* gene expression shows a significant relationship with *ZAP70* gene and TTFT in independent CLL cohorts. A similar correlation between high DDR1 expression and worse prognosis has been reported in different solid tumours, where DDR1 has been involved in interaction between stroma and tumour cells. Indeed, DDR1 acts as a sensor for various types of collagen of the extracellular matrix, and mediates enhanced tumour cell migration, survival, proliferation and matrix remodelling.^2^ Therefore, DDR1 has a pro-invasive role in different tumours and is studied as a target for kinase inhibitors. In AML, DDR1 responds to remodelled type IV collagen present in the stroma of the bone marrow microenvironment.^4^ It is conceivable that DDR1 may act as a sensor for stromal collagen of the bone marrow and lymphoid tissues also in CLL and provide a supportive stimulus, acting in concert with other environmental signals. Indeed, DDR1 may act in concert with other receptor systems. For example, the Insulin-like growth factor-I (IGF-I)/IGF-I receptor system cooperates with DDR1 and the G-protein oestrogen receptor to support progression, in mesothelioma and lung cancer cells.^15^ In normal T cells, DDR1 is expressed only upon cell activation through mechanisms, which involve ERK1/2 signalling. Similarly, normal circulating B cells or tonsil germinal centre B cells do not express DDR1,^5^ suggesting that stimuli activating the ERK/1/2 pathway may induce DDR1 expression in CLL cells. However, the mechanisms controlling DDR1 expression in CLL remain elusive, since we could not find any correlation between DDR1 expression and constitutive activation of the ERK1/2 pathway. Also, different miRNA have been reported to regulate DDR1 expression, such as miR199b-5p in AML cells,^4^ or IL-21-induced miR663b in B-CLL cells.^8^ Nonetheless, the combined analysis of DDR1 gene and miRNA expression profiles failed to identify significant anti-correlations suggestive of miRNA regulating DDR1 expression, in B-CLL cells. In conclusion, the correlation of *DDR1* mRNA levels with CLL outcome and other biomarkers of progression suggests a potential role of DDR1 in CLL biology and lends support to further studies to address the functional role of DDR1 in this disease.

## Figures and Tables

**Figure 1:**
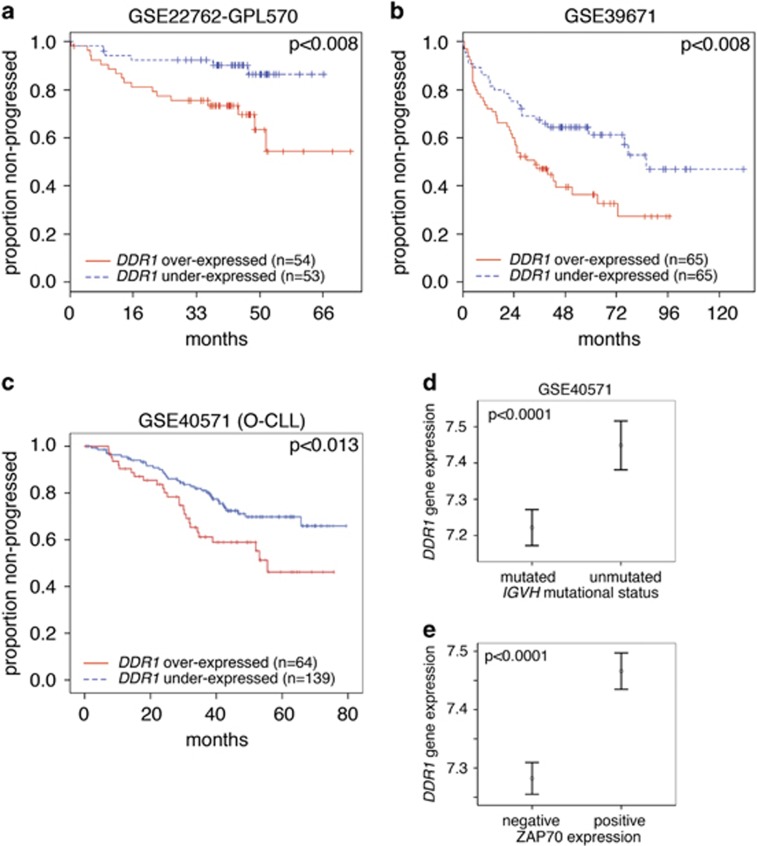
(**a–c**) High *DDR1* gene expression correlates with a shorter time-to-treatment in CLL in three public-access data sets. Cut-off was median *DDR1* value in **a** and **b**. In **c** the best cut-off point for *DDR1* gene expression discriminating cases with immunoglobulin variable heavy *(IGVH)* mutated from those unmutated was sought by constructing receiver operating character (ROC) analysis. Curves were constructed using the Kaplan–Meier method and compared with the Wilcoxon log-rank test. (**d**, **e**) *DDR1* gene expression is higher in CLL with unmutated *IGVH* relative to mutated cases (**d**) and in ZAP70-positive cases relative to negative ones (**e**) (mean±s.e.m.).

**Figure 2:**
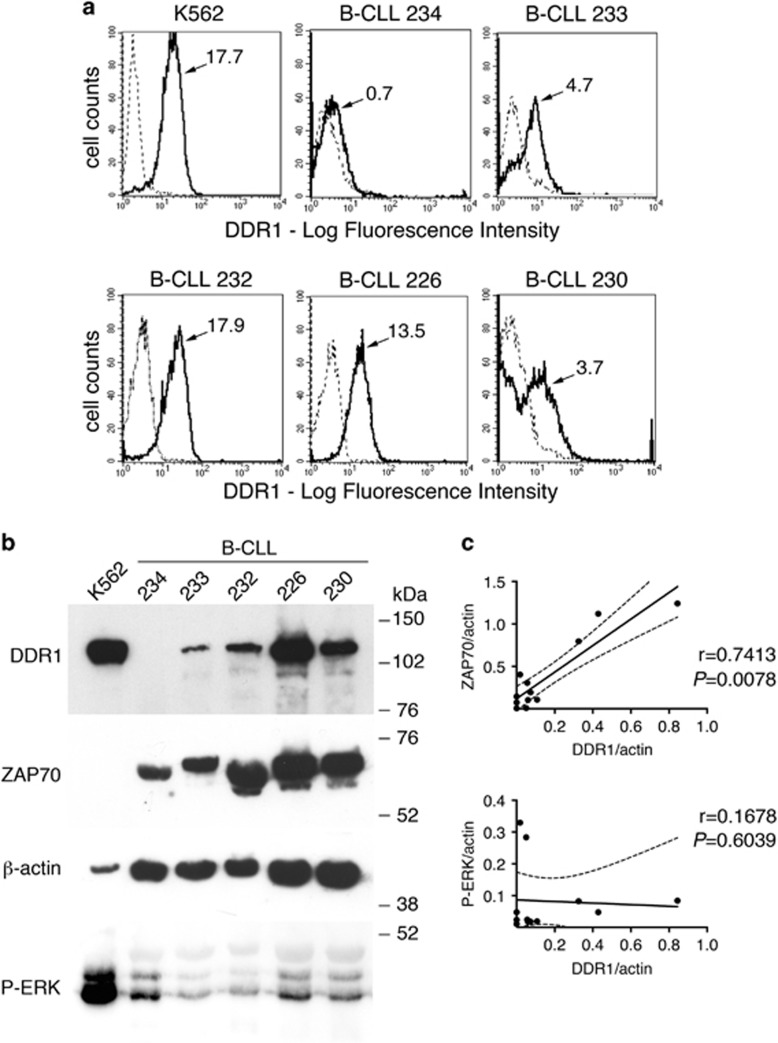
(**a**) Peripheral blood mononuclear cells from CLL cases express variable levels of surface DDR1 as detected by immunofluorescence and fluorescence-activated cell sorter analysis, gated on lymphoid cells; (**b**) western blot analysis of DDR1, ZAP70 and pERK1/2: DDR1 showed a predominant band of an apparent mw of ~130 kDa. K562 erythroleukemia cell line is shown as positive control (**c**) Densitometry analyses of ZAP70 and DDR1, relative to actin control, showed a significant correlation, whereas no correlation with phosphorylated ERK1/2 (P-ERK) was found (analysis includes also western blots shown in Supplementary Figure S2).
