# Supplementary material for: Heterogeneous expression of the collagen receptor DDR1 in chronic lymphocytic leukaemia and correlation with progression
Source: Blood Cancer J. 2017 Jan 6;7(1):e513–. doi: 10.1038/bcj.2016.121 (PMC5301030; doi:10.1038/bcj.2016.121)
Supplement: Supplementary Information [file bcj2016121x1.pdf]

Supplementary information

Figure S1

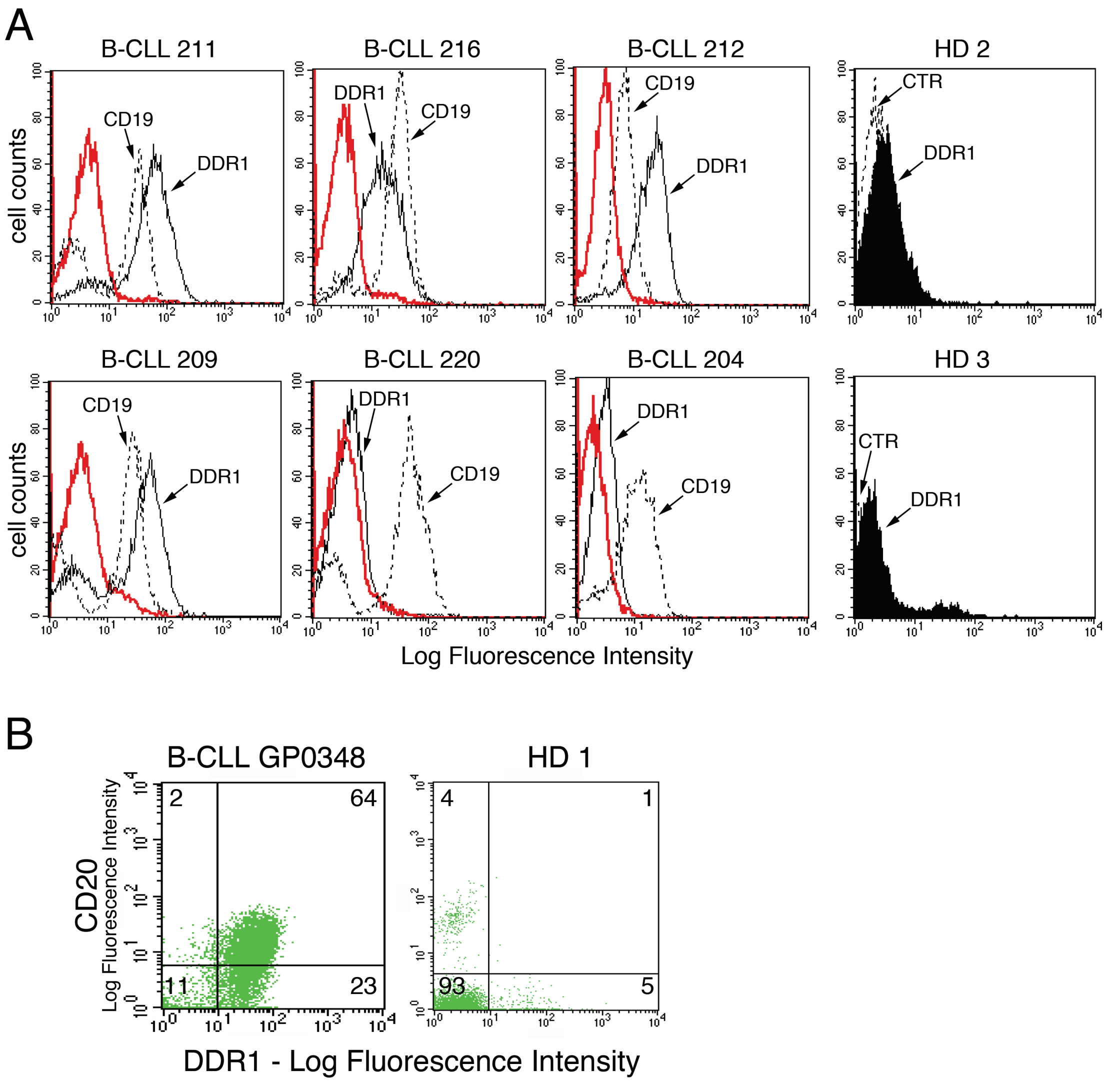

Supplementary Figure 1: Heterogeneity of surface DDR1 protein expression levels detected by indirect immunofluorescence.

A) PBMC from CLL cases express variable levels of surface DDR1 (black line) as detected by indirect immunofluorescence and FACS analysis. Red line is the negative control using secondary reagent only. The dotted line shows reactivity with a FITC-labelled anti-CD19 mAb (cat N. 21270193 Immuno Tools, Friesoythe, Germany). Six CLL cases representative of 34 analysed are shown. Two healthy donors (HD) are also shown.

B) Two colour immunofluorescence with anti-DDR1 and anti-human CD20 mAbs shows double staining in CLL cells. A representative CLL case and a healthy donor (HD) are shown. For two colours immunofluorescence, indirect immunofluorescence with anti-DDR1 murine mAb and AlexaFluor488-labelled anti-IgG1 secondary antibody was combined with a PE-labelled mouse IgG2b Anti-Human CD20 (Clone 2H7, cat. N.555623, Becton Dickinson Italia, Milan, Italy).

Figure S2

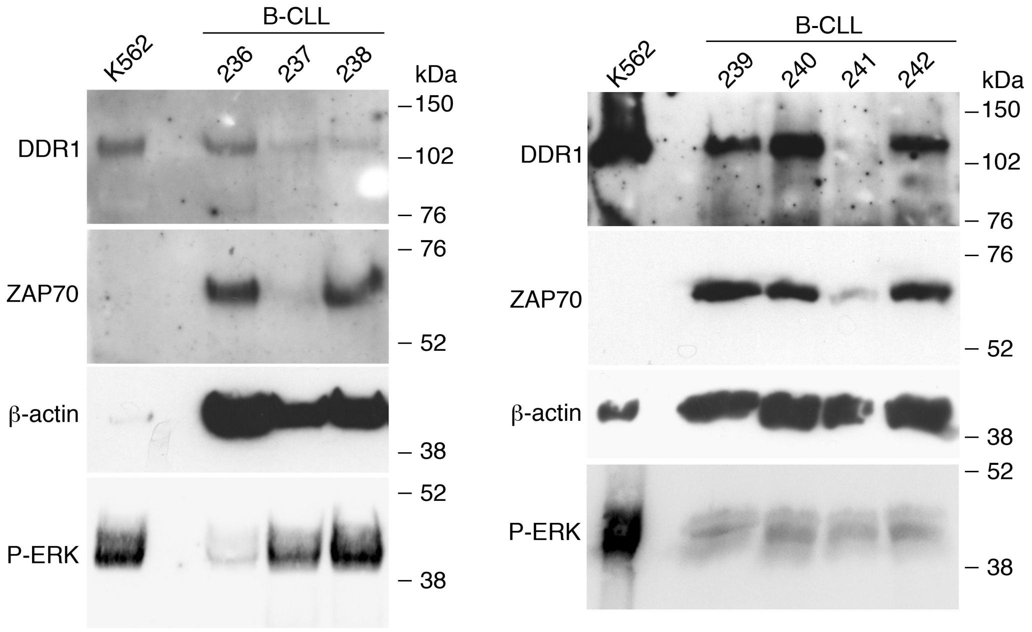

Supplementary Figure 2: Western blot analysis of DDR1, ZAP70 and P-ERK1/2 in 7 additional CLL cases. K562 erythroid leukemia cell line is shown as positive control and β-actin as loading control. Before re-probing, blots were stripped with Restore Western Blot Stripping Buffer (Thermo Fisher Scientific, Life Technologies Italia, Monza, Italy). CLL cells were lysed in 10 mM Tris-HCl pH 7.5, 1 mM EDTA, 150 mM NaCl, 1% Triton-X-100, 0.1% SDS, 1 mM sodium orthovanadate and 1 mM PMSF. Lysates were resolved by 10% SDS-PAGE under reducing conditions and analysed by Western blotting using the following antibodies: rabbit anti-DDR1 (Cell Signaling Technology, Danvers, MA), mouse anti-ZAP70 (clone 2F3.2, Upstate, Merck Millipore, Vimodrone, Italy), anti-phospho-ERK1/2 (Santa Cruz Biotechnology, Heidelberg, Germany) and anti-β actin (Sigma-Aldrich, Milan, Italy), according to manufacturers' instructions. Proteins were detected by ECL Prime (GE Healthcare Italia, Milan, Italy) and autoradiography.

Table S1. Characteristics of the CLL cases analysed by Western blot.

B-CLL case n.	CD38	IgGVH
**226**	2[a)]	unmut
**230**	8	unmut
**232**	2	unmut
**233**	78	unmut
**234**	5	NA [b)]
**236**	35	NA
**237**	40	unmut
**238**	7	unmut
**239**	80	unmut
**240**	NA	mut
**241**	NA	mut
**242**	50	unmut

a) % positive cells, flow cytometry
b) NA = not available

Table S2. Characteristics of the CLL cases analysed by immunofluorescence.

	N. of cases
Total	34
ZAP70[a)]	
≥ 30	10
< 30	13
NA[b)]	11
CD38	
≥ 30	5
< 30	20
NA	9
IGVH status	
mutated	14
un-mutated	11
NA	9

[a)] % positive cells, flow cytometry
[b)] NA not available
